# Efficient Genome Editing in Chicken DF-1 Cells Using the CRISPR/Cas9 System

**DOI:** 10.1534/g3.116.027706

**Published:** 2016-02-10

**Authors:** Yichun Bai, Linjie He, Pengcheng Li, Kun Xu, Simin Shao, Chonghua Ren, Zhongtian Liu, Zehui Wei, Zhiying Zhang

**Affiliations:** College of Animal Science and Technology, Northwest A&F University, Yangling, Shaanxi, 712100, China

**Keywords:** CRISPR/Cas9 system, surrogate reporter, chicken DF-1 cell, mutant efficiency, off-target, GenPred, Shared data resource, Genomic Selection

## Abstract

In recent years, genome engineering technology has provided unprecedented opportunities for site-specific modification of biological genomes. Clustered regularly interspaced short palindromic repeats (CRISPR)/CRISPR-associated (Cas) 9 is one such means that can target a specific genome locus. It has been applied in human cells and many other organisms. Meanwhile, to efficiently enrich targeted cells, several surrogate systems have also been developed. However, very limited information exists on the application of CRISPR/Cas9 in chickens. In this study, we employed the CRISPR/Cas9 system to induce mutations in the peroxisome proliferator-activated receptor-γ (*PPAR*-γ), ATP synthase epsilon subunit (*ATP5E*), and ovalbumin (*OVA*) genes in chicken DF-1 cells. The results of T7E1 assays showed that the mutation rate at the three different loci was 0.75%, 0.5%, and 3.0%, respectively. In order to improve the mutation efficiency, we used the *Puro^R^* gene for efficient enrichment of genetically modified cells with the surrogate reporter system. The mutation rate, as assessed via the T7E1 assay, increased to 60.7%, 61.3%, and 47.3%, and subsequent sequence analysis showed that the mutation efficiency increased to 94.7%, 95%, and 95%, respectively. In addition, there were no detectable off-target mutations in three potential off-target sites using the T7E1 assay. As noted above, the CRISPR/Cas9 system is a robust tool for chicken genome editing.

In recent years, genome engineering technologies have provided unprecedented opportunities for site-specific modification of biological genomes. These technologies could be used to investigate the function of coding genes, or regulatory elements, via gene editing (Li *et al.* 2015; Wanzel *et al.* 2016). The most important component of these technologies is a nuclease that can introduce double-strand breaks (DSBs) into specified regions of genomes. Representative examples of this technology, *e.g.*, engineered zinc-finger nuclease (ZFN), transcription activator-like effector nuclease (TALEN), and clustered regularly interspaced short palindromic repeats (CRISPR)/CRISPR-associated (Cas)9, have developed rapidly in recent years.

CRISPR/Cas9 derives from bacterial and archaeal adaptive immune systems that defend against invasion by phages or foreign plasmids (Barrangou *et al.* 2007). The CRISPR/Cas9 system can target a specific genome locus by using a Cas9 protein and a guide RNA (gRNA), which includes a 20 nt sequence that binds to its DNA target by Watson-Crick base-pairing (Jinek *et al.* 2012). The target site must have a sequence motif, known as the protospacer adjacent motif (PAM), present just downstream of the 20 bp target sequence (Jinek *et al.* 2012). Unlike ZFN and TALEN, which require engineering of a new protein for each target sequence, the only required engineering in the CRISPR/Cas9 system is to match a 20 nt target-complementary gRNA with the target DNA sequence adjacent to the PAM. As such, these gRNAs can be rapidly constructed and are easy to use. After *in vitro* work showed the site-specific cleavage function (Jinek *et al.* 2012), the CRISPR/Cas9 system was promptly developed. To date, it has been applied in human cells, and in many other organisms (Mali *et al.* 2013a; Cong *et al.* 2013), such as zebra fish (Yin *et al.* 2015; Li *et al.* 2015), mice (Miura *et al.* 2015; Mou *et al.* 2015), rats (Guan *et al.* 2014; Li *et al.* 2013), pigs (Zhou *et al.* 2016; Ruan *et al.* 2015), and goats (Wang *et al.* 2015). However, there is little information about the application of this technology in chicken (Veron *et al.* 2015).

Not all designed nucleases are efficient enough to allow sufficient derivation of cells containing nuclease-driven mutations (Santiago *et al.* 2008; Kim *et al.* 2009). Laborious screening of many clones is often required to obtain enough gene-modified clones. Kim and colleagues devised several surrogate reporters that contained a nuclease target sequence to enrich gene-modified clones, and eliminate unmodified cells (Kim *et al.* 2011, 2009, 2013). In our previous work, we developed a dual reporter system for efficient enrichment of genetically modified cells (Ren *et al.* 2015). Here, we demonstrate efficient site-specific modification of the peroxisome proliferator-activated receptor-γ (*PPAR*-γ), ATP synthase epsilon subunit (*ATP5E*), and ovalbumin (*OVA*) genes in chicken DF-1 cells using the CRISPR/Cas9 system combined with a dual surrogate reporter system. The results indicate that this system is a robust tool for chicken genome editing.

## Materials and Methods

### Construction of the Cas9 nuclease expression vector

The CRISPR/Cas9 system used in this study was the engineered *Streptococcus thermophilus* CRISPR3-Cas (StCas9) system constructed by Xu *et al.* (2015). The human codon-optimized *Cas9* gene and the gRNA were driven by the CMV and U6 promoters, respectively, and cloned into the pll3.7 vector. The targeting oligonucleotide sequences used for the respective gRNAs were: *PPAR*-γ, GCGAATGCCACAAGCGGAGA; *ATP5E*, GCCTCAGTACAAAGCTGAGG; and *OVA*, AGATGTTCTCATTG GCATGG.

### Construction of the surrogate reporter

**Figure 1:**
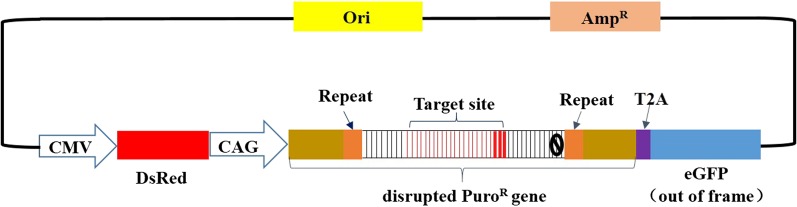
Schematic of the SSA-RPG reporter. The *DsRed* is driven by the CMV promoter. The *Puro^R^* gene is interrupted by a target sequence flanked with direct repeats as SSA arms. The disrupted *Puro^R^* and *eGFP* genes are fused by *T2A*, as a dual reporter, and driven by the CAG promoter.

Three target sequences of approximately 170 bp in length for the *PPAR*-γ, *ATP5E*, and *OVA* genes were PCR-amplified using the chicken genome as a template using the primers shown in Table 1. Reference genomic sequences were extracted from GenBank (*PPAR*-γ, NC_006099; *ATP5E*, NC_006107; and *OVA*, NC_006089). Then, the target sequence was inserted into the parental DsRed-Puro^R^-eGFP (RPG) dual-reporter surrogate system based on the single strand annealing (SSA) repair pathway (SSA-RPG) (Ren *et al.* 2015) to generate corresponding reporter vectors. The surrogate SSA-RPG reporter vector included three reporter genes: *DsRed*, *Puro^R^*, and *eGFP*. *DsRed* was driven by a CMV promoter to measure transfection efficiency. The *Puro^R^* gene was driven by a CAG promoter, and fused with the e*GFP* gene via T2A as a dual-reporter. The CRISPR/Cas9 target sequence was flanked with 200 bp direct repeats, and inserted into the middle of the *Puro^R^* gene to interrupt the open reading frame (ORF) (Figure 1). When the CRISPR/Cas9 nuclease cleaves the target site of the surrogate reporter to introduce the DSBs, repair via SSA between the two direct repeats can result in correction of the ORF for the *Puro^R^* and *eGFP* reporter genes.

**Table 1:** Primer sequences for generating RPG reporter plasmid

Gene	Forward Primer	Reverse Primer
*PPAR*-γ	CCCgcggccgc TTCTTCAGCCATCAGGTTTGGG	CCCggatcc CCTTGGCTTTGGTCAGAGGG
*ATP5E*	CGCgcggccgc GTTTTCAGCTACATCCGGTACTC	CGCggatcc CCTTCTTGGTCTTCACAATCTTC
*OVA*	CCCgcggccgc AGAGTTCACCATGGGCTCCATC	CCCggatcc GTATACCATGGCTAGAGCTGAC

### Culture of DF1 cell line

The chicken DF-1 cell line was maintained in Dulbecco’s modified Eagle medium (DMEM, Gibco) containing 10% (v/v) fetal bovine serum (FBS, Sciencecell), 100 U/ml penicillin, and 100 μg/ml streptomycin in an incubator at 37° and 5% CO_2_. DF-1 cells were seeded into 24-well plates, and transfected 24 hr later.

### Cell transfection

Cells were cotransfected with 1.6 μg plasmid DNA containing Cas9/gRNA, and the corresponding SSA-RPG reporter plasmid (molar ratio 1:1) using Sofast transfection reagent (Xiamen Sunma Biotechnology Co., Ltd. China), according to the manufacturer’s protocol. The Cas9 expression vector alone, with the SSA-RPG reporter, were used as controls. The cells were observed and photographed with a fluorescence microscope 2 d after transfection. Then, half of the cells were harvested for genome detection, and the other half for puromycin screening.

### Puromycin selection

At 2 d after transfection, puromycin (2.5 μg/ml) (Sigma) was added to the culture medium and maintained for 4 d; the medium was changed daily. Then, the puromycin was removed, and the resistant cells were cultured sequentially until 90% confluence for subsequent genome detection.

### Detection of nuclease-induced mutations

Genomic DNA was isolated from harvested DF-1 cells with the phenol-chloroform method. A T7E1 assay was performed as previously reported (Kim *et al.* 2009; 2011). PCR products were amplified using the primer pairs listed in Table 2, and purified by gel extraction. We denatured 100 ng purified product at 94°, annealed it to form heteroduplex DNA, subsequently treated it with 5 U of T7 nuclease I (New England Biolabs) for 30 min at 37°, and finally analyzed it using 2% agarose gel electrophoresis. Mutation frequencies were calculated as previously described based on the band intensities using ImageJ software and the following equation: mutation frequency (%) = 100 × [1 – (1 – F)^1/2^], where F represents the cleavage coefficient, which is the proportion of the total relative density of the cleavage bands to all of the relative densities of the cleavage bands and uncut bands (Guschin *et al.* 2010).

**Table 2:** Primers used in sequence analysis for detection of indels

Gene	Forward Primer	Reverse Primer	Product Size
*PPAR*-γ	AAGCGCTTCAGTAGTTTGCC	TTGTGGAAGATAACCTCTGG	673 bp
*ATP5E*	CATGGTGGCGTACTGGCGGCAGGC	TGAGCTGCTCGCTGCATGTGCAGTG	546 bp
*OVA*	TAGCCTACCATAGAGTACCCTG	CAACTGCTGGATGCAGAGCACTAGC	697 bp

To further confirm target locus mutations, PCR products were cloned into the pGEM-T Easy vector (Promega). For each sample, 19–20 random clones were sequenced to identify the mutation efficiency.

### Off-target analysis in the DF-1 cell line

Three potential CRISPR/Cas9 off-target sites for the *PPAR*-γ, *ATP5E*, and *OVA* genes were selected for mutation analysis (Table 3). Off-target analysis was performed by T7E1 assay. Primers for the amplification of nine potential target fragments are listed in Table 3. Reference genomic sequences were obtained from GenBank (GNS, NC-006088; EHD3, NC-006090; ADCYAP1, NC-006089; KCNT2, NC-006095; DAD1, NC-006114; PITPNM2, NC-006102; ABCC9, NC-006088; TXNDC12, NC-006095; and LCP2, NC-006100).

**Table 3:** Primer sequences for detection of off-target analysis

Gene	Forward Primer	Reverse Primer	Product Size
*GNS*	AGCCCCACAGATAGCTGTC	TTCACGCCAGCCACTCCTTC	544 bp
*EHD3*	GAGGTTGTGACTCAGTCAGCAC	CACATACATTATCCGAGCCCG	627 bp
*ADCYAP1*	GAAATGGAGCAAACAGAGAG	CGTCCTTCATTTGTACTCAGG	626 bp
*KCNT2*	CCTCCCCAACAATCACCTCTTCCC	GCTGGGGAAGAAGCAGACA	610 bp
*DAD1*	CTCACACAAGGGCACCTCTG	GATAGCTACGGGGCTTCGTG	636 bp
*PITPNM2*	GCTGGATCTGTGCATACAAG	GGGTTCATACATGCCATGAC	597 bp
*ABCC9*	GGCTACTTGGGTACTGCACTC	CAGTTGCTGCAAAGATCACGC	388 bp
*TXNDC12*	CATGCTGACCCGGAAGTGAC	CCGTACACTGATTGATGTGGTG	363 bp
*LCP2*	ACCTGAGCCAAGCCTGACTC	TAGGGAGCTGGATTCATTTTCC	387 bp

### Data availability

The authors state that all data necessary for confirming the conclusions presented in the article are represented fully within the article.

## Results

### Detection of the CRISPR/Cas9 activity in DF-1 cells

**Figure 2:**
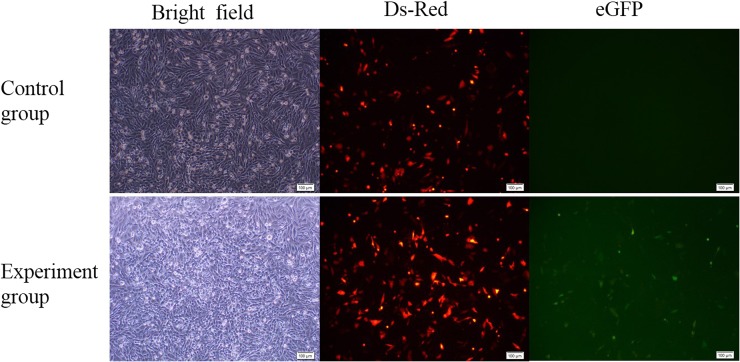
Activity detection of the CRISPR/Cas9 system 48 hr after transfection. Visualization of DsRed and eGFP expression by fluorescence microscopy after transfection for 48 hr. Cells from the experimental group expressed DsRed and eGFP, but the control group expressed DsRed only.

**Figure 3:**
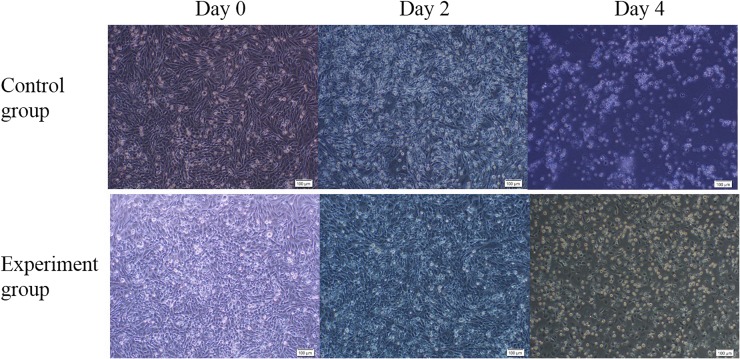
T7E1 assay for the indels induced by CRISPR/Cas9 within unselected and puromycin-selected cells. (A), (B), and (C) represent the indels induced by PPAR-γgRNA/Cas9, ATP5E.gRNA/Cas9, and OVA.gRNA/Cas9, respectively. The numbers at the bottom of the gel indicate the mutation frequency as measured by band relative density.

Three genes, *PPAR*-γ, *ATP5E*, and *OVA*, were selected as the target genes. To quickly test the activity of the CRISPR/Cas9 system, all Cas9 and gRNA plasmids, combined with the corresponding SSA-RPG reporter, were cotransfected into DF-1 cells in the experimental groups, with the Cas9 expression vector without gRNA being used in the control groups. At 48 hr after transfection, a considerable fraction of cells expressed Ds-Red, and a small portion of cells expressed eGFP in the experimental group, whereas cells in the control group expressed only Ds-Red. As expected, all of the eGFP-expressing cells also expressed Ds-Red in the experimental group (Figure 2). This indicated that the CRISPR/Cas9 system worked in DF-1 cells. The subsequent T7E1 assay demonstrated that the mutation frequencies within the *PPAR*-γ, *ATP5E*, and *OVA* loci were 0.75%, 0.5%, and 3.0%, respectively (Figure 3).

### Mutant cell enrichment using puromycin screening

**Figure 4:**
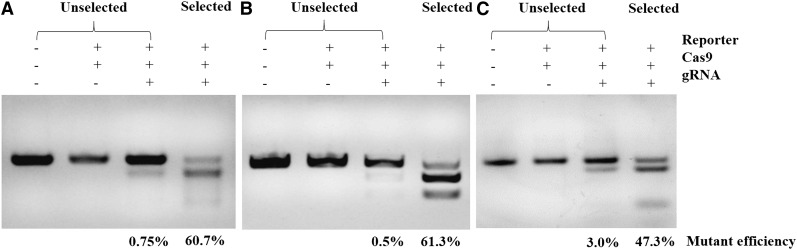
Puromycin sensitivity after puromycin was added. Most of the cells from the control group died, and a portion of cells in the experimental group were alive after puromycin addition for 2 and 4 d. These results indicated that the CRISPR/Cas9 system worked in DF-1 cells. The control group shows cells transfected with Cas9 only and the corresponding RPG reporter. The experimental group refers to cells transfected with CRISPR/Cas9 and the corresponding RPG reporter.

**Figure 5:**
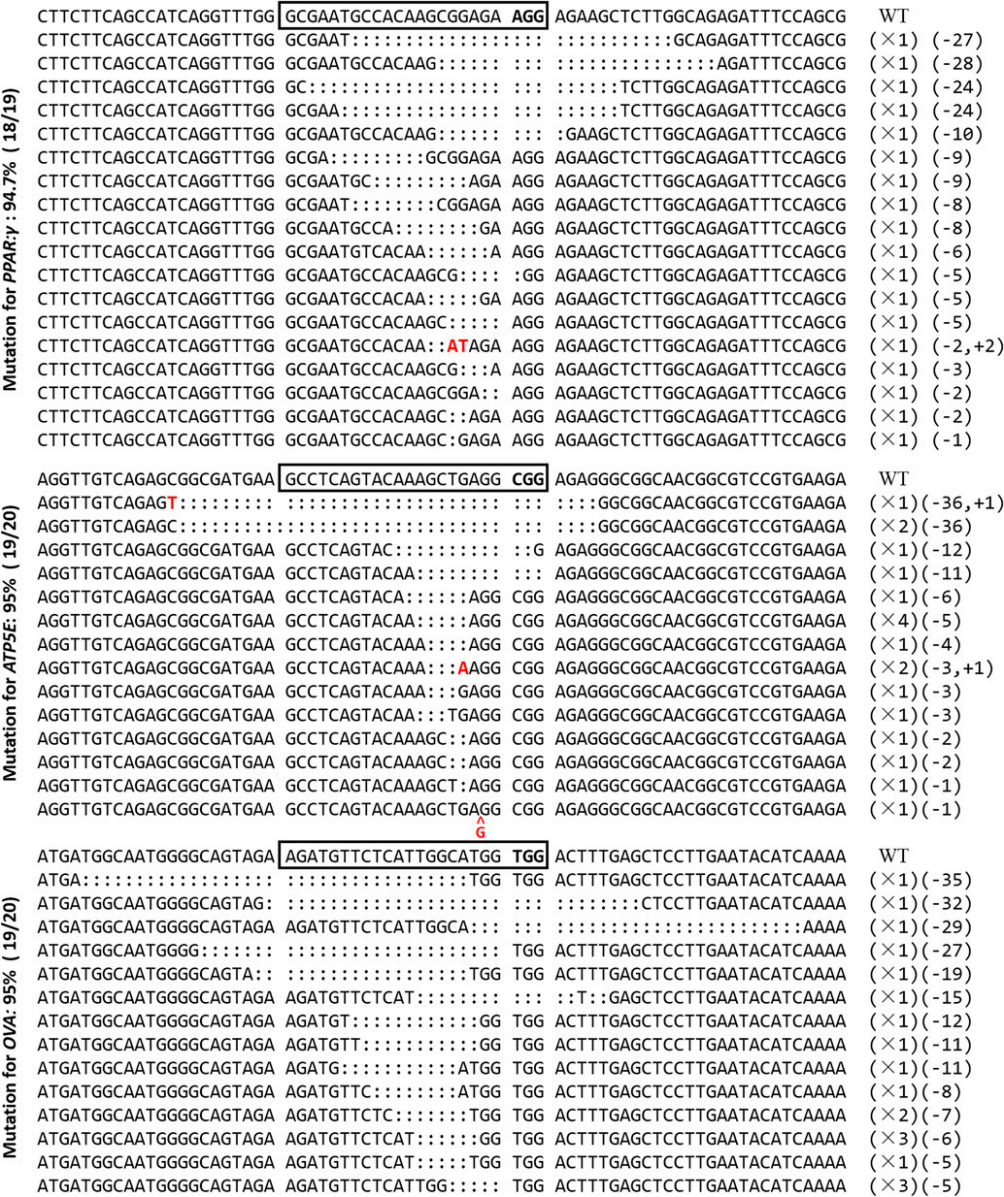
Sequencing results of mutations induced by CRISPR/Cas9 combined with an SSA-RPG reporter based on puromycin-screened cells. Boxes indicate target sites for CRISPR/Cas9 system. Dashes and red letters indicate deleted and inserted base pairs. X1, X2 ,X3, and X4 indicate the number of each clone.

**Figure 6:**
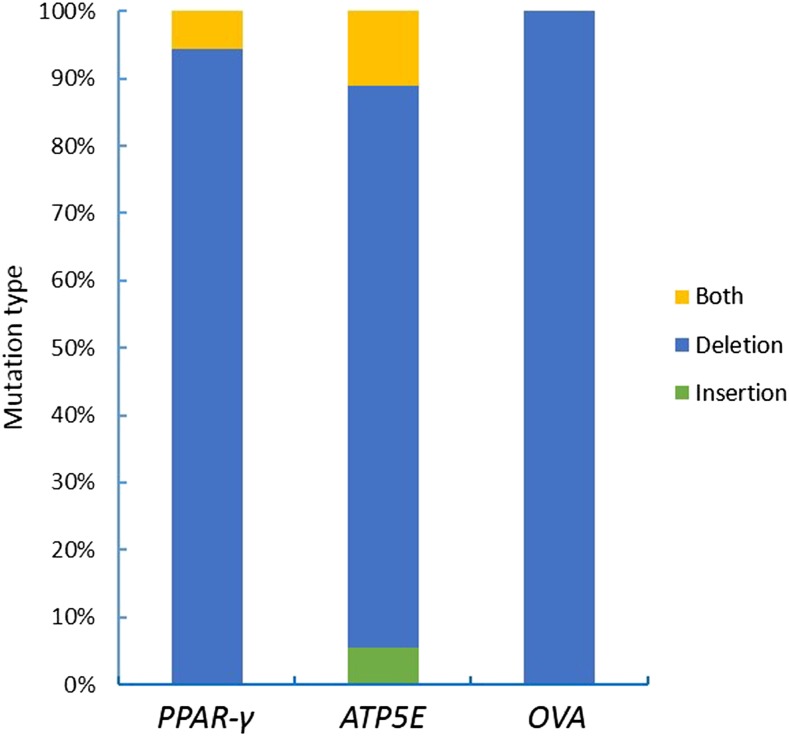
Mutation types in DF-1 cells induced by CRISPR/Cas9 combined with the SSA-RPG reporter.

In order to obtain more mutant cells, we used the *Puro^R^* gene for fast and efficient enrichment of genetically modified cells. Puromycin was added to the medium of the experimental group and the control group; 4 d later, most of the cells in the control group were dead, and a fraction of the cells were alive in the experimental group (Figure 4). The remaining cells in the experimental group were harvested for sequencing analysis. T7E1 assay showed mutation frequencies within the *PPAR*-γ, *ATP5E*, and *OVA* loci for the screening cells of 60.7%, 61.3%, and 47.3%, respectively (Figure 4). However, since the T7E1 assay tends to underestimate fold enrichment (Kim *et al.* 2011), we subsequently sequenced the mutation site by cloning the PCR products in the T vector. The results showed different mutations at three target sites; efficiencies of up to 95% were observed for the three genes (Figure 5). This showed that the mutation efficiency was improved greatly by puromycin screening with the surrogate reporter. Most of the mutations were deletions, with only a few mutations being insertions, or both deletion and insertion (Figure 6). These data demonstrated that the selected gRNAs, combined with the surrogate reporter, worked efficiently in chicken genomes, and that deletion was the main type of mutation.

### Detection of an off-target effect

**Figure 7:**
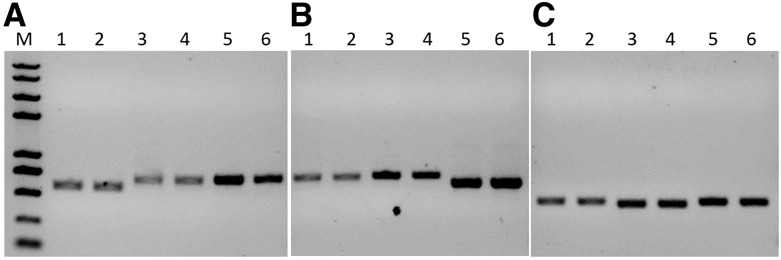
T7E1 assay of nine potential off-target effects. (A) Off-target effect for PPAR-γ.CRISPR/Cas9. One, 3, and 5 indicate the control group without puromycin screening for *GNS*, *EHD3*, and *ADCYAP1*, respectively. Two, 4, and 6 indicate the experimental group with puromycin screening for *GNS*, *EHD3*, and *ADCYAP1*, respectively. (B) Off-target effect for ATP5E.CRISPR/Cas9. One, 3, and 5 indicate the control group without puromycin screening for *KCNT2*, *DAD1*, and *PITPNM2*, respectively. Two, 4, and 6 indicate the experimental group with puromycin screening for *KCNT2*, *DAD1*, and *PITPNM2*, respectively. (C) Off-target effect for OVA.CRISPR/Cas9. One, 3, and 5 indicate the control group without puromycin screening for *ABCC9*, *TXNDC12*, and *LCP2*, respectively. Two, 4, and 6 indicate the experimental group with puromycin screening for *ABCC9*, *TXNDC12*, and *LCP2*, respectively (M, Plus2k marker).

To test whether an off-target effect occurred in these puromycin-resistant cells, we predicted a total of nine potential off-target sites for *PPAR*-γ, *ATP5E*, and *OVA*. There were no detectable off-target mutations in these loci using the T7E1 assay (Figure 7).

## Discussion

CRISPR/Cas9 depends on small RNAs for sequence cleavage, and only a programmable RNA is required to generate sequence specificity (Jinek *et al.* 2012). Therefore, CRISPR/Cas9-mediated genome engineering is easy to handle, highly specific, efficient, and multiplex (Mali *et al.* 2013a). It has been used widely in many organisms and cells since the demonstration of the site-specific cleavage function *in vitro* (Mali *et al.* 2013b; Cong *et al.* 2013). Here, we showed that the CRISPR/Cas9 system combined with a surrogate reporter can target chicken DF-1 cells efficiently.

The CRISPR/Cas9 system used in this study was from *S. thermophilus* (Xu *et al.* 2015), and the *Cas9* gene in this system was codon humanized. The stCRISPR/Cas9 system provided the ability to cleave the target site effectively in chicken DF-1 cells. Véron and colleagues efficiently targeted the *PAX7* gene in the chicken embryo using mammalian codon-optimized Cas9 (Véron *et al.* 2015), thus indicating the high similarity of codon usage between mammals and chickens. However, the mutation efficiency was very low according to T7E1 assay analysis. Therefore, we needed to remove the unwanted cells from the total cells so that we could enrich for the mutant cells. CRISPR/Cas9-modified cells can be enriched by selecting puromycin-resistant cells using a single vector expressing puromycin (Shalem *et al.* 2014; Yuen *et al.* 2015; Ran *et al.* 2013b). However, the activity of the nuclease cannot be assured using this single vector system, since this method enriches the transfected cells. In our surrogate system, the disrupted *Puro^R^* gene, fused with *eGFP* gene of the SSA-RPG reporter, would be corrected via the SSA repair mechanism when the CRISPR/Cas9 nuclease cleaves the target site of the surrogate reporter. So, the puromycin-resistant cells would be eGFP^+^ cells. A target sequence on a surrogate reporter could reflect nuclease activity in the same cell (Kim *et al.* 2011, 2014; Ramakrishna *et al.* 2014; Ren *et al.* 2015). Therefore, we could monitor CRISPR/Cas9 activity in live cells by fluorescent microscopy. Furthermore, enrichment can also be achieved with fluorescence activated cell sorting (FACS) by evaluating the fraction of eGFP^+^ cells alongside puromycin screening. The repeat length around the SSA-RPG surrogate reporter used in this reporter was 200 bp because the repair efficiency is much higher for the SSA-RPG reporter with a 200 bp repeat length (Ren *et al.* 2015). Surprisingly, it was found that the mutation efficiency increased to approximately 95% for these genes in the DF-1 cell line after puromycin screening. This was higher than the mutation efficiency observed in HEK293T cells (Ren *et al.* 2015). There are several probable reasons for the high efficiency using this system in DF-1 cells. First, DF-1 cells grow more slowly than HEK293T cells, so the Cas9 nuclease could work effectively in almost every CRISPR/Cas9 transfected DF-1 cell. Second, the SSA-mediated repair efficiency in the DF-1 cell line was probably higher than that in HEK293T cells, so we obtained more puromycin-resistant cells. Third, the large T-antigen of simian virus 40 (SV40) gene probably could influence the expression of CRISPR/Cas9 because HEK293T cells are stably transfected with the large T-antigen gene of SV40, while the DF-1 cell line was developed spontaneously from fibroblasts of chicken embryo (Himly *et al.* 1998). Among the mutations observed, deletion was the main type. These results are consistent with those found in transgenic chickens induced by TALEN (Park *et al.* 2014), and somatic cells induced by the CRISPR/Cas9 nuclease (Veron *et al.* 2015). However, the mechanisms require further study.

DNA damage can cause erroneous changes in the genetic code, leading to increased mutation load or wider-scale genome aberration that can threaten cell or organism viability (Jackson and Bartek 2009; Roos and Kaina 2013). So, off-target mutations remain a major concern for CRISPR/Cas9 (Cho *et al.* 2014; Tsai *et al.* 2015; Fu *et al.* 2013; Pattanayak *et al.* 2013; Veres *et al.* 2014; Zhang *et al.* 2015) because it is likely to cause unwanted chromosomal rearrangements (Cho *et al.* 2014). The potential off-target effect must not be ignored. Several methods could reduce off-target effects, such as considering the tolerance of mismatches and PAM mutations (Fu *et al.* 2013), controlling the dosage of CRISPR/Cas9 (Hsu *et al.* 2013; Pattanayak *et al.* 2013), and using paired Cas9 nickase (Shen *et al.* 2014; Ran *et al.* 2013a), or truncated gRNA (Fu *et al.* 2014). In this study, like most of the studies cited above, an off-target effect was not detected in the selected potential off-target sites by T7E1 assay. But T7E1 assay cannot detect off-target mutations that occur at frequencies < 1% due to its poor sensitivity (Kim *et al.* 2009). In order to detect off-target effect more reliably, several other methods, such as deep sequencing (Cho *et al.* 2014), high-throughput genomic translocation sequencing (HTGTS) (Frock *et al.* 2015), Genome-wide Unbiased Identification of DSBs Enabled by Sequencing (GUIDE-seq) (Tsai *et al.* 2015), etc. could be used.

In conclusion, we showed that the CRISPR/Cas9 system, combined with an SSA-mediated surrogate reporter, can efficiently target chicken DF-1 cells. This will help to study the function of chicken genes both efficiently and cheaply.
